# Empathy enhances decoding accuracy of human neurophysiological responses to emotional facial expressions of humans and dogs

**DOI:** 10.1093/scan/nsae082

**Published:** 2024-11-07

**Authors:** Miiamaaria V Kujala, Lauri Parkkonen, Jan Kujala

**Affiliations:** Department of Psychology, Faculty of Education and Psychology, University of Jyväskylä, PO Box 35, Jyvaskyla FI-40014, Finland; Faculty of Veterinary Medicine, University of Helsinki, PO Box 57, Helsinki FI-00014, Finland; Department of Neuroscience and Biomedical Engineering, Aalto University School of Science, PO Box 12200, Espoo FI-00076, Finland; Department of Neuroscience and Biomedical Engineering, Aalto University School of Science, PO Box 12200, Espoo FI-00076, Finland; Department of Psychology, Faculty of Education and Psychology, University of Jyväskylä, PO Box 35, Jyvaskyla FI-40014, Finland

**Keywords:** facial expression, emotion, object perception, magnetoencephalography, event-related potential, machine learning

## Abstract

Despite the growing interest in the nonhuman animal emotionality, we currently know little about the human brain processing of nonconspecific emotional expressions. Here, we characterized the millisecond-scale temporal dynamics of human brain responses to conspecific human and nonconspecific canine emotional facial expressions. Our results revealed generally similar cortical responses to human and dog facial expressions in the occipital cortex during the first 500 ms, temporal cortex at 100–500 ms and parietal cortex at 150–350 ms from the stimulus onset. Responses to dog faces were pronounced at the latencies in temporal cortices corresponding to the time windows of early posterior negativity and late posterior positivity, suggesting attentional engagement to emotionally salient stimuli. We also utilized support vector machine-based classifiers to discriminate between the brain responses to different images. The subject trait-level empathy correlated with the accuracy of classifying the brain responses of aggressive from happy dog faces and happy from neutral human faces. This result likely reflects the attentional enhancement provoked by the subjective ecological salience of the stimuli.

## Introduction

Domestic dog (*Canis familiaris*) is the first domesticated animal (Larson et al. 2014), today forming mixed-species social groups with human families. Dogs are involved in many kinds of cooperations with humans—e.g. in agility sports, scent detection, or as service dogs. This kind of cooperation requires some interpretation of another species, and human social cognition appears to partially extend to nonconspecifics (Harrison and Hall 2010). As face perception gives rise to an experience of agency (Broadbent et al. 2013), it is important to comprehend whether the basic neural processing of facial expressions extends from human to dog faces. Recent scientific studies have pioneered this direction (Blonder et al. 2004, Spunt et al. 2017, Bunford et al. 2020), but we still know little about the human neural processing of the canine companions.

Human brain processing of conspecific faces has been extensively mapped with functional magnetic resonance imaging (fMRI; for reviews, Haxby et al. 2000, Kanwisher and Yovel 2006). Similarly to human faces, dog faces activate the human brain regions within the lateral fusiform gyrus and inferior occipital gyrus (Blonder et al. 2004, Bunford et al. 2020, Boch et al. 2023). However, activation in the medial fusiform gyrus—or functionally localized fusiform face area—is often stronger for human than dog faces (Blonder et al. 2004, Boch et al. 2023; but see Bunford et al. 2020). Also, amygdala and posterior superior temporal gyrus show stronger activation for human versus dog faces (Blonder et al. 2004, Bunford et al. 2020). Human and nonhuman faces also trigger similar emotional attribution (Spunt et al. 2017). Generally, adult humans detect the valence of dog facial expressions likewise to those of humans (Schirmer et al. 2013, Kujala et al. 2017), and human brain activation to emotional human faces differs from dog faces only within superior temporal sulcus (Spunt et al. 2017). However, as the focus has been on haemodynamics rather than electrophysiology, the fast dynamics of these neural responses is not known.

Affective processing guides attention towards ecologically relevant stimuli in the environment (for reviews, Pourtois and Vuilleumier 2006, Olofsson et al. 2008, Wieser et al. 2014). Generally, negative visual stimuli may amplify early processing, whereas stimulus arousal affects processing at the later stages (Olofsson et al. 2008). Negative or threatening conspecific faces enhance early visual responses at 90–110 ms (Halgren et al. 2000, Pourtois et al. 2004), possibly reflecting rapid modulation of primary visual cortex by feedback from emotion-related subcortical regions (Anderson and Phelps 2001, Amaral et al. 2003, Vuilleumier et al. 2004). Threatening emotional stimuli further enhance subsequent, attention-driven processing of non-emotional visual targets in lateral occipital regions peaking at 135 ms (Pourtois et al. 2004, 2005, Rellecke et al. 2012). Instead, the face-sensitive response peaking approximately at 170 ms in the occipito–temporal regions (Bentin et al. 1996, Kanwisher et al. 1997) appears less sensitive to the emotional content (Streit et al. 2000, Sato et al. 2001, Balconi and Pozzoli 2003).

Subsequent brain electrophysiological responses, early posterior negativity (EPN) and late posterior positivity (LPP) are modulated by the interaction of emotion and task (Schindler et al. 2020, Schupp and Kirmse 2021). EPN is a sustained deflection provoked by affective stimuli, detectable at temporo-occipital EEG sensors and peaking at 200–350 ms (Schupp et al. 2004). Such long-latency responses are detected for emotional stimuli such as picture assemblies (Bekhtereva et al. 2015) and facial expressions of fear, anger, pain, or happiness (Kujala et al. 2009, Calvo and Beltrán 2013, Sawada et al. 2014, Yoon et al. 2016, Schindler et al. 2020). Observation of angry human facial expressions enhances the response amplitude of all the abovementioned processing stages irrespective of the task, even with corresponding low-level visual properties (Rellecke et al. 2012). However, no evidence exists on whether threatening facial expressions of nonconspecifics such as dogs provoke similar responses as those of conspecific humans.

Empathy is divided into cognitive and emotional subparts (Davis 1980), and subject empathic abilities are related to their visceral responses (De Vignemont and Singer 2006). Observing affective images of dogs results in brain activation of areas associated with emotional empathy (Franklin et al. 2013), suggesting the extension of emotional empathy to nonconspecifics. Empathy also appears to enhance electrophysiological brain responses during observation of emotional stimuli (Choi et al. 2014). More empathic individuals are quicker and more accurate in evaluating the emotion from human facial expressions (Besel and Yuille 2010, Kosonogov et al. 2015) and show enhanced facial mimicry (Rymarczyk et al. 2016). More empathic subjects also rate threatening facial expressions of both humans and dogs more quickly and strongly, while empathy increases the ratings of positive expressions only for conspecific humans (Kujala et al. 2017). As emotional empathy directed toward humans and animals are linked (Paul 2000), these results suggest a more general role of emotional empathy in processing both human and nonhuman expressions.

Classifying observed visual stimuli from the brain activity was first performed in fMRI studies differentiating low-level stimulus properties (Kamitani and Tong 2005, Haynes and Rees 2006) and image categories, including faces (Haxby et al. 2001, Carlson et al. 2003, Cox and Savoy 2003). In electrophysiology, decoding brain activity with machine-learning algorithms has also utilized the available temporal information (Carlson et al. 2013, Ramkumar et al. 2013). Classification studies have dissociated the neural processing of animate versus inanimate as well as conspecific versus nonconspecific image categories (Contini et al. 2017), and shown that attention enhances the prediction accuracy of objects from nonobject stimuli (Carlson et al. 2003). Recently, individual differences between subjects, such as personality traits, have been differentiated based on spatial configurations of fMRI data in idle tasks (Dubois et al. 2018, Hsu et al. 2018, Jiang et al. 2018, Misra et al. 2021). However, how factors such as empathy contribute to the classification accuracy of individual neurophysiological responses is largely unknown.

Our main aim was to characterize the temporal dynamics of human neurophysiological responses to conspecific human and nonconspecific dog emotional facial expressions, and to examine the accuracy of classifying the brain responses with machine-learning approaches. We were interested in the accuracy of differentiating dog and human facial expressions (happy, neutral, aggressive) from the brain responses; if the classification of dog and human facial expressions follow similar patterns; and whether biologically relevant threat-processing of human and dog faces occurs alike. Finally, we examined the subjective contribution for the success of differentiating between emotional face stimuli: How does subject trait-level empathy contribute to the individual variability in success of the classification accuracy?

## Methods

### Ethics statement

The experimental protocols of the study were approved by the Aalto University Research Ethics Committee (Board Meeting 6 March 2014). Participants gave their informed consent prior to the experiment, and all methods were performed in accordance with the relevant regulations.

### Subjects

Subjects were 15 healthy volunteers, aged 28 ± 4 years (mean ± SD; 8 F/7 M). All had normal or corrected-to-normal vision, and all subjects were right-handed according to the Edinburgh Handedness Inventory (Oldfield 1971). Six subjects had lived in a family with a pet dog, and 3/15 had some experience as a dog handler through hobbies (eg dog shows, hunting). The subjects had thus relatively low expertise of dog behavior.

### Stimuli

Eight different categories of stimuli were obtained from our previous study with dogs (Kujala et al. 2020). The stimuli were color photographs of faces [threatening/aggressive dogs/humans (AD/AH), neutral dogs/humans (ND/NH), and pleasant/happy dogs/humans (HD/HH), household objects (OB) and phase-scrambled images (S; Supplementary Fig. S1)]. The low-level visual properties of the face stimuli versus objects, or emotional expression categories did not differ, but human faces differed from dog faces, as previously reported (Kujala et al. 2020). For details of the face stimuli, see Somppi et al. (2016); for objects, Stacy and colleagues (1997) and for phase-scrambled images, Kujala et al. (2017).

Additionally, the color stimuli of previous studies were transformed to grayscale versions, and the low-level visual properties (spectrum, histogram, and intensity) of the grayscale images were equalized with the SHINE toolbox in Matlab (Willenbockel et al. 2010). Therefore, two sets of stimuli were used in the experiment: original color stimuli and the grayscale stimuli with equalized low-level properties.

### Experimental procedure

The study comprised (i) simultaneous electroencephalography (EEG) and magnetoencephalography (MEG) acquisition with an acquisition protocol as closely matching our previous experiment with noninvasive dog EEG (Kujala et al. 2020) as possible; (ii) a behavioral measurement with stimulus emotional rating and behavioral questionnaire, and (iii) an anatomical T1-weighted MR image. Behavioral, EEG/MEG and MR measurements were scheduled on different days; EEG/MEG acquisition preceded the behavioral measurement.

### Behavioral questionnaires and stimulus rating

Subjects completed the Big Five Inventory sampling personality (John and Srivastava 1999); the Interpersonal Reactivity Index sampling trait-level empathy (IRI, Davis 1980); and animal-directed IRI (Norring et al. 2014) sampling empathy for animals. IRI is divided in four factors: two cognitive empathy factors *Perspective-taking* (PT) and *Fantasy Scale*, and the emotional empathy factors *Emotional Concern* (EC) and *Personal Distress*. On the basis of previous literature (Besel and Yuille 2010, Kujala et al. 2017), we focused on the cognitive empathy factor PT and the emotional empathy factor EC as the main features affecting emotion detection from both human and animal-directed IRI. The IRI samples the trait-level empathy, but for simplicity, we henceforth refer to it with the term “empathy”. The subjects also rated the amount of valence, arousal, and six discrete emotions on a 7-point scale for each stimulus image, while their responses and the response times were recorded. Statistical analyses of the behavioral data are shown in Supplementary Materials.

### Neurophysiological EEG/MEG and anatomical data acquisition

EEG and MEG data were acquired simultaneously in a magnetically shielded room in the MEG Core of Aalto Neuroimaging Infrastructure, Aalto University. EEG was acquired with an EEG cap with 32 Ag/AgCl electrodes, placed according to the international 10/20 system; MEG was acquired with the 306-channel whole-head Elekta Neuromag™ Vectorview MEG system (MEGIN Oy, Helsinki, Finland). The data were filtered to 0.03–200 Hz and sampled at 600 Hz. The head position was measured at the beginning of each measurement; additionally for two subjects, continuous head tracking was used due to the low quality of the initial head position measurement. In the subsequent analyses, we focus mainly on the MEG data.

During the EEG/MEG data acquisition, the stimuli were projected on a back-projection screen located 1.23 m in front of the subject. The stimulus presentation was controlled with Presentation® software (http://nbs.neuro-bs.com/). Stimuli were overlaid on a gray background on a screen area of 47.6 cm × 26.8 cm, and the stimulus images were on average 23 cm × 24.5 cm in size at the center of the presentation screen. Stimuli were presented in four consecutive presentation sequences, with short breaks between the sequences. Half of the sequences contained the color images and other half the grayscale versions; the presentation order of the stimulus sequences was counterbalanced.

The sequences started with a fixation cross in the middle of the screen. The stimuli were shown in blocks of 15–19 images, with duration of 500 ms per stimulus and a 500–1500 ms interstimulus-interval; the interval between the stimulus blocks was 5 s, during which a text “Break” was shown. Each of the 80 different stimuli were repeated 8–10 times resulting in 176 stimuli per category (88 color + 88 grayscale images).

Standard anatomical T1-weighted MR images were acquired in the Advanced Magnetic Imaging Centre of Aalto Neuroimaging Infrastructure, Aalto University; three sets of MRIs were obtained from previous research, with the permission of the subjects.

### MEG data preprocessing

MEG data were preprocessed with MNE Python (Gramfort et al. 2013). First, sensor-level noise was suppressed with the Oversampled Temporal Projection method (Larson and Taulu 2018). External disturbances were further removed using the spatiotemporal signal space separation method (Taulu and Simola 2006) that was used also to transform the subjects’ heads to a common position to facilitate group-level analysis of sensor-level data. Last, independent component analysis (Hyvärinen 1999) was used to suppress ocular and cardiac artifacts.

### Sensor-level analysis

Sensor-level averaged evoked responses were calculated across both color and grayscale images within each stimulus category in the time window of −200 to 500 ms with respect to stimulus onsets. The responses were baseline-corrected (baseline time window −200 to 0 ms) and low-pass filtered at 40 Hz. Category-specific areal averages of the evoked responses were computed across the 204 gradiometers across 12 different sensor groups (12–20 sensors per group; left/right frontal, central, parieto-occipital, occipital, and both anterior and posterior temporal cortex). Vector sums were computed for each gradiometer pair before averaging the signals within regions.

### Source modeling of evoked responses

The cortical sources of the evoked responses were estimated with MNE (Gramfort et al. 2014). Cortically constrained noise-normalized L2-minimum-norm estimates were obtained in eight 100 ms-long time windows between 50 and 500 ms in a regularly spanned grid consisting of ∼4700 points across subjects. The noise covariance matrix used in the estimation was obtained from empty room recordings, and fixed source orientations were used in the source modeling. The source-level responses were baseline-corrected (baseline −200 to 0 ms) and low-pass filtered at 40 Hz. Z-scores at each time point and source location were computed by dividing the responses with the standard deviation within the baseline time-window. The individual-level *Z*-scores were transformed to a template anatomy (Fischl et al. 1999) and averaged across the 14 subjects for whom the source-modeling could be performed successfully. The group-level *Z*-scores were visualized using Freesurfer 5.3 (Fischl 2012). Additionally, response strengths for human faces were compared with dog faces (see Supplementary Material).

### Analysis with machine learning

Machine-learning-based classification analyses were conducted using the Statistics and Machine Learning Toolbox in Matlab R2020b. Two different analyses were performed across the color and grayscale stimuli utilizing support vector machine (SVM) classifiers, separately for MEG and EEG data. First, time-resolved binary classification was performed in 80 ms time-windows with 50% overlap between 0 and 500 ms. The individual trials were baseline-corrected (baseline time window −200 to 0 ms) and low-pass filtered at 40 Hz. Separate SVM classifiers were trained for each binary classification task across all pairs of categories, that is (7 × 8)/2 = 28 classifiers for each subject and time-window. For MEG and for each classification, those 50 sensors were selected that showed the highest inter-trial synchrony across the trials and categories in the time window 0–500 ms. This approach allows reducing the number of features to be reasonable by focusing the analysis to sensors with stable neural response patterns outweighing noise in the data (Mitchell et al. 2008). In the EEG classification, data from all 32 sensors were used. Before applying the SVM classifiers, the data were vectorized into 2450-dimensional (49 time points × 50 MEG channels) and 1568 feature vectors (49 time points × 32 EEG sensors) within each time-window. A linear kernel was used in the SVM, and a 5-fold cross-validation was applied to estimate the accuracy of the classification. Before dividing the data into the different folds, the order of the trials was shuffled to avoid using sequential data samples in the training and testing data. The classification accuracies for each binary comparison were determined by averaging the classification accuracies across the different fold combinations (four folds used for training, one for testing).

The significance of the classification was determined via a permutation-based maximum statistics approach. The category labels of the data we randomly permuted 200 times prior to training and testing and a null distribution was generated from the obtained classification accuracies. A maximum-statistics approach was applied simultaneously across all time-windows and category-pairs to determine the 95% confidence limit, corresponding to *P* < .05 (corrected across all comparisons). SVM-based classification was also performed using all time points within the 0–500 ms time-window. The procedure was otherwise identical with the time-resolved classification, except that for this static classification the feature vector had 15 050 (301 time points × 50 MEG channels) or 9632 dimensions (301 time points × 32 MEG channels) and that here we averaged three separate trials together to increase the signal-to-noise ratio of the training and testing data samples.

### Examination of classification accuracy

Classification accuracy between stimulus categories in the 500 ms time-window was first divided into three classes: ≥ 90% accuracy (excellent); ≥ 70% accuracy (good) and 60–70% accuracy (fair). The classification pairs with good or fair accuracy were examined for the dependency of the categories with subject individual differences in empathic reactivity (Davis 1980) and/or behavioral response times in rating the stimulus valence/arousal.

Good/fair classification pairs with angry/aggressive expressions (AD and AH) as well as happy human expressions (HH) were included in the empathic reactivity analyses due to the correlation of their valence/arousal rating with EC and/or PT (Kujala et al. 2017). Thus, the correlation of subject EC and PT with the classification accuracy between the pairs AD versus HD; AD versus ND; AH versus. HH; AH versus NH and HH versus NH was examined with Spearman’s rho using a bootstrapping procedure (1000 samples, bias corrected and accelerated); for dog expressions, also ani-EC and ani-PT was calculated. As empathy affects the emotion detection from human faces (Besel and Yuille 2010, Kosonogov et al. 2015), the correlation of subject response times in stimulus valence/arousal rating with the good/fair classification accuracy of human expressions was examined with Spearman’s rho using bootstrapping procedure (1000 samples, bias corrected and accelerated).

## Results

### Behavioral rating and response times

Subjects’ evaluation of the valence, arousal, and six emotions in each of the stimuli, and statistical analysis of valence/arousal ratings, are shown in Supplementary Materials and Table S1. Supplementary Fig. S2 shows the results generally following the stimulus behavioral ratings of a previous study (Kujala et al. 2017) in a separate sample.

Average response times for each stimulus category are shown in Table 1. As the questions were presented in a fixed order, the response times between questions are not comparable; however, the response times between the stimulus categories can be compared. Response times differed in evaluating the valence of facial expressions [χ^2^(5) = 12.20, *P* = .032; Friedman and *W* = 0.174, Kendall]. Pairwise comparisons showed that the differences in response times originated in the human expressions; responses were quicker to HH versus AH as well as to NH versus AH (*z* = −3.3, *P* < .001; *z* = −2.3, *P* = .02, respectively). Response times in evaluating the arousal of human and dog stimuli did not differ between categories [χ2(5) = 3.39, *P* = .640; Friedman].

**Table 1.** Subject response times for each stimulus category (in seconds; mean ± SEM).

	HH	HD	NH	ND	AH	AD	OB	SD
Valence	14.1 ± 1.5	16.3 ± 1.3	16.2 ± 1.7	16.6 ± 1.8	19.6 ± 2.3	16.9 ± 2.4	10.0 ± 1.4	6.2 ± 0.6
Arousal	12.1 ± 2.0	11.7 ± 1.4	10.6 ± 1.2	10.1 ± 1.0	10.7 ± 1.0	11.3 ± 1.6	8.2 ± 1.4	5.7 ± 0.8
Happiness	8.2 ± 0.7	8.1 ± 0.9	8.0 ± 0.9	7.0 ± 0.8	6.0 ± 1.0	5.6 ± 0.7	3.9 ± 0.8	2.3 ± 0.4
Sadness	5.3 ± 0.5	5.9 ± 0.6	8.7 ± 0.9	7.4 ± 0.8	7.1 ± 1.0	6.9 ± 0.8	3.0 ± 0.5	2.3 ± 0.3
Surprise	6.8 ± 0.6	6.9 ± 0.6	6.0 ± 0.6	5.8 ± 0.6	8.0 ± 0.7	8.6 ± 0.8	3.8 ± 0.8	2.2 ± 0.4
Disgust	5.0 ± 0.6	5.9 ± 0.8	6.7 ± 0.9	5.1 ± 0.4	9.5 ± 0.9	8.1 ± 0.9	3.2 ± 0.7	2.5 ± 0.3
Fear	5.8 ± 0.9	6.3 ± 0.9	8.1 ± 1.0	7.3 ± 1.0	9.3 ± 0.9	9.9 ± 1.4	2.7 ± 0.5	2.3 ± 0.4
Anger	4.6 ± 0.5	5.4 ± 0.6	7.3 ± 0.9	6.4 ± 0.8	8.3 ± 0.7	8.1 ± 1.3	3.3 ± 0.7	2.4 ± 0.3

### Event-related responses

**Figure 1.**
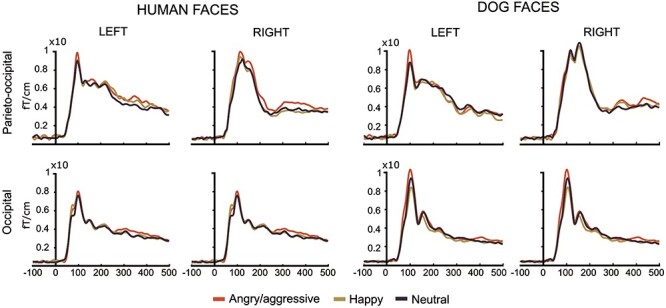
Group-level event-related brain responses plotted as areal averages of sensor-level MEG data (vector sums of each planar gradiometer pair) over the left and right parieto-occipital and occipital regions.

Figure 1 shows the sensor-level MEG responses for the selected cortical regions to the human and dog angry/aggressive, happy, and neutral facial expressions, averaged across participants. All face stimulus categories showed the highest amplitude responses at 105–110 ms and sustained response strengths above the baseline level until the end of the examined time-interval, with subtle variations between the stimulus categories.

### Cortical sources

**Figure 2.**
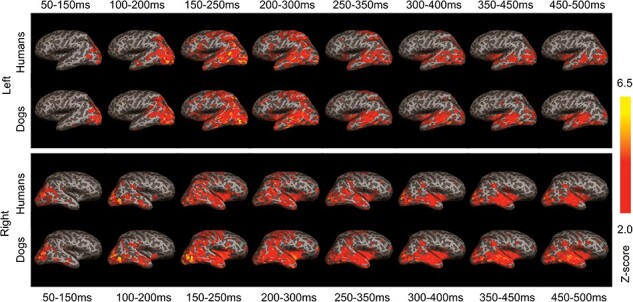
Temporal progression of the grand-averaged event-related MEG responses to dog and human faces are shown from 50 ms of the onset, averaged over 100 ms with a 50 ms overlap, visualized at the level of the cortical current sources. Top: left hemisphere, bottom: right hemisphere.

Figure 2 shows the group-level cortical responses averaged across different facial expressions for human and dog stimuli in eight different 100 ms time windows. Overall, the cortical responses were similar for human and dog faces, with neural activity in the bilateral occipital cortex throughout the 500 ms time window, in the bilateral parietal cortex ∼150–350 ms, and in the bilateral temporal cortex ∼100–500 ms. Statistical comparison between human faces versus dog faces are shown in Supplementary Fig. S3.

### Machine-learning analysis

**Figure 3.**
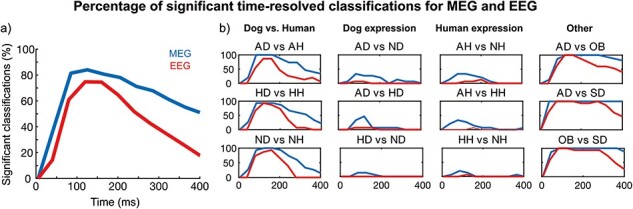
Percentage of significant classification results in different 80 ms time windows illustrating the successful classification utilizing the time-resolved information (a) across all condition pairs and (b) in different comparison pairs of dog versus human facial expressions, dog facial expressions, human facial expression, and other comparisons.

Figure 3 shows the results of the time-resolved classification. For each time-window, the percentage of significant (*P* < .05, corrected for multiple comparisons) pairwise classifications was calculated across the 28 category pairs. In the MEG analysis, significant classifications were detected after 50 ms with the highest percentage of classifications occurring between 100 and 150 ms. The percentage of significant classifications remained at ∼60% until the latest 80 ms window (Figure 3a). The time-resolved classifications were successful especially for comparisons across species and against the object and scrambled images, whereas the success rate was lower for the within-species comparisons (see Figure 3b). The EEG analyses revealed qualitatively similar findings but with lower levels of significant classifications. For EEG, the maximum classification percentage did not reach as high levels and the percentage tended to drop faster towards the end of the examined time-interval compared to MEG.

**Figure 4.**
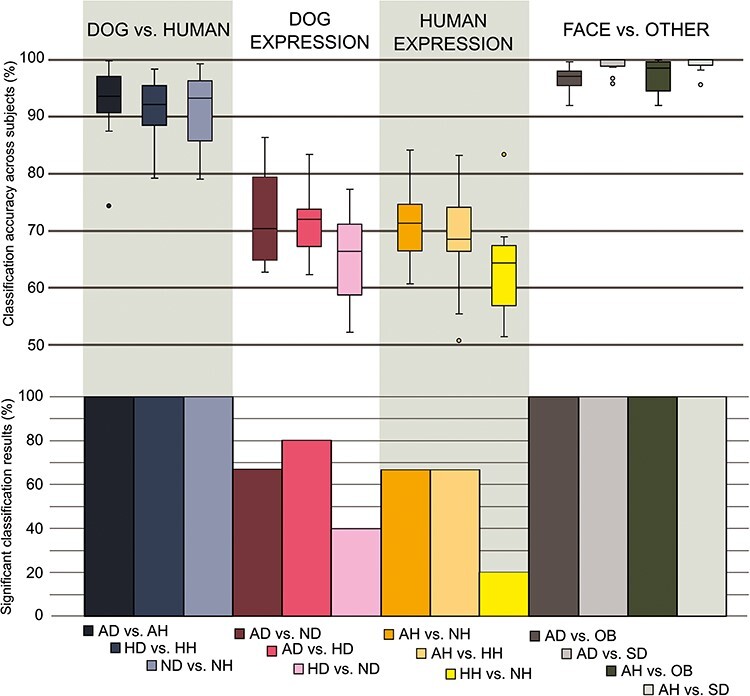
Top: Classification accuracy across subjects as a boxplot in pairwise comparisons of dog versus human, dog facial expression, human facial expression; and faces (aggressive dog/human) versus objects or scrambled images. Bottom: Percentage of subjects with significant classification results in the pairwise comparisons. The pairs are given below the figure, background shadowing differentiates dog versus human comparisons; comparisons of dog emotional expressions; comparisons of human emotional expressions and face versus control image comparisons.

For the MEG data, discrimination of event-related brain responses over the whole 0–500 ms time window was successful in 20–100% of the subjects between all stimulus categories (see Figure 4), and discriminating the scrambled images from any other stimulus category yielded the highest accuracies. Discrimination of all face categories versus scrambled was >99% (across-subjects range 94–100%; and for object versus scrambled 98% (range 95–100%). Discrimination of human faces from dog faces with comparable valence also yielded excellent accuracy of 93–91% (range 74–100%). The EEG classification findings across the 0–500 ms time window are shown in Supplementary Materials (Supplementary Fig. S4).

Classification accuracy of MEG data across subjects in comparisons of species, emotions, or objects is depicted in the top part of Figure 4. Classification accuracy between the different facial expressions within species was either good or fair and it followed a similar pattern in both species. Aggressive expressions were best discriminated from neutral or happy expressions; discrimination of happy from neutral expressions of both species yielded only fair level of accuracy. Between dog expressions, the classification accuracy was as follows: AD versus ND 72% (across-subject range 63–86%), AD versus HD 71% (range 62–83%), and HD versus ND 65% (range 52–77%). Between human expressions, the accuracy was the following: AH versus NH 70% (range 60–84%), AH versus HH 69% (range 51–83%), and HH versus NH 63% (range 51–83%). These classification accuracies were significant (*P* < .05, corrected for multiple comparisons) in all 15 subjects when comparing facial stimuli across species or between human or dog faces and objects or scrambled images (see Figure 4 bottom).

### Association of classification accuracy with behavioral variables

**Figure 5.**
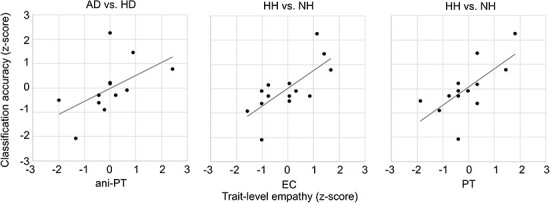
Correlation of subject trait-level empathy scores with the classification accuracies between aggressive versus happy dog expressions (AD versus HD; MEG) and human happy versus neutral expressions (HH versus NH; EEG). Correlations are *z*-scored data utilizing comparable scales for visualization.

Figure 5 shows the correlation of subject empathy with the classification accuracies between AD versus HD and HH versus NH. Both EC and PT correlated with the EEG classification accuracy between HH versus NH; animal-directed PT also correlated with AD versus HD, whereas AD versus ND, AH versus HH or AH versus NH did not correlate with empathy scores (Supplementary Table S2).

Examination of the classification accuracy between the human facial expression categories with respect to the subject response times revealed the connection of AH versus HH classification accuracy with the response times of AH valence and HH arousal (Supplementary Table S3).

## Discussion

### Behavioral and brain processing of human and dog facial expressions

Our primary goals were to characterize the temporal dynamics of human brain responses to human and dog emotional facial expressions, and to examine the accuracy of classifying these responses with machine learning. Human and dog facial expressions followed similar behavioral evaluations: happy faces were rated as most positive, whereas angry/aggressive faces provoked the most emotional arousal, followed by happy and neutral faces, in line with previous data (Schirmer et al. 2013, Kujala et al. 2017, Törnqvist et al. 2023). Also, the brain results are concordant with the previous fMRI studies examining nonconspecific affective expressions (Franklin et al. 2013, Spunt et al. 2017). Starting at 50 ms after the stimulus onset, the brain responses to human and dog facial expressions followed a similar temporo–spatial pattern.

The machine learning-based classification results aligned with our previous experiment with dogs (Kujala et al. 2017). Differentiation of the responses was most successful for visually most differing categories (faces versus non-faces), and the informative time windows were largely similar, with most significant results occurring at around 100–150 ms and 200–300 ms. Previously, classification of human electrophysiological responses to human facial expressions of differing valence have been successful in the frequency domain (Li and Lu 2009). Here, face versus nonface and human versus. dog comparisons during the first 500 ms were the most successful, and responses to angry/aggressive versus. other expression categories were also differentiated satisfactorily. However, distinguishing brain responses to happy versus neutral expressions of either species yielded lower accuracy—classification of the EEG data was close to chance level. This may reflect the contribution of the later processing stages, from 200 ms onward, to the static classification, as the angry/aggressive faces were rated higher in arousal than other expressions in both species.

### Processing of threat from human and dog faces

We were also interested in the threat-processing related to the human and dog facial expressions. Emotion, attention, and image properties have interconnected effects within different stages of visual processing, with image properties having greater effects on the early responses and emotional attention modulating the later responses (Pourtois et al. 2013, Schindler et al. 2018). Therefore, perhaps through multiple additive effects, angry human faces elicit larger neural responses starting at 50 ms (Rellecke et al. 2012). Consistent with previous work, we observed high-amplitude responses for both human and dog angry/aggressive facial expressions at the parietal and parieto–occipital sensors from 100 ms onwards. Previously, the canine ERPs were likewise pronounced to facial expressions of threat (Kujala et al. 2020).

Here, the electrophysiological brain responses to dog faces appear pronounced also in temporal cortices at 200–500 ms, corresponding to the time windows of EPN and LPP (for a review, Schupp et al. 2006). These later processing stages are connected to highly arousing emotional content, including threatening animals (Schupp and Kirmse 2021) and correspond to early attentional selection (Schupp et al. 2004). These late processing stages are also largely independent of other low-level properties (Schettino et al. 2016, Schindler et al. 2018) than the stimulus size, which may recruit stronger arousal (Codispoti and De Cesarei 2007). Thus, as ecologically salient stimuli, the threatening (angry/aggressive) expressions likely cause sustained attention that is reflected in the late brain responses.

### Subject emotional empathy enhances decoding accuracy of emotional visual stimuli

Empathy has been associated with faster and more accurate detection of human expressions (Besel and Yuille 2010, Kosonogov et al. 2015, Kujala et al. 2017) and with rating threatening human and dog faces, together with happy human faces, higher in valence and/or arousal (Kujala et al. 2017). Subjective relevance of threatening stimuli also facilitates attention and behavioral responses (Öhman et al. 2001). Here, we asked whether this kind of subjective experience affects the machine learning-based classification of brain responses.

Our results show that the brain responses of subjects with higher trait-level emotional empathy yielded higher classification accuracy for differentiating aggressive from happy dog faces and happy from neutral human faces, with strong or moderate correlation between empathy and classification accuracy (Cohen 1988). As emotional arousal affects the electrophysiological brain responses related to the early selection of attention (Schupp et al. 2004, Pourtois et al. 2013, Schindler et al. 2018), and, in turn, increased attention facilitates the classification of visual stimuli (Carlson et al. 2003), our current results likely reflect attentional enhancement provoked by the subjective ecological salience of the stimuli.

The results are consistent with findings that images high in emotional arousal amplify the attention-related brain responses within the first 500 ms regardless of the valence (Schupp and Kirmse 2021). As the stimuli of aggressive dogs and happy humans have received the highest arousal ratings and correlated with subject EC and PT (Kujala et al. 2017), the current results may reflect the differential emotional reactivity of the subjects, captured by the trait-level empathy. The classification accuracy of aggressive versus other human faces also correlated with the response times, suggesting that these images provoked more subjective evaluation. Generally, happy human faces are recognized faster than neutral faces (Leppänen and Hietanen 2004), and emotional empathy may further strengthen the recognition (Besel and Yuille 2010). As emotions have widespread effects in the visual areas through the bidirectional connections with amygdala (Vuilleumier et al. 2001, Amaral et al. 2003, Pourtois et al. 2013), empathy-mediated attentional recruitment appears to strengthen the differentiation between ecologically salient stimuli during the first 500 ms of the response.

Early attentional modulation of brain responses by ecologically salient stimuli through the subcortical magnocellular pathway is well established (Vuilleumier 2005, Pessoa 2008). However, the effect of subjective appreciation of the stimulus salience, modulated by empathy, on the classification of brain responses has not been reported before. The correlation of empathy and decoding accuracy was conducted with bootstrapping, but it is noteworthy that the current sample size may pose restrictions to the generalizability of the results. Thus, similar studies aiming to improve our understanding of brain decoding at individual-level are needed.

After the suggestion of subjective mind-reading by Kamitani and Tong (2005), brain decoding has focused on classifying perceptual responses with high degree of similarity across subjects (for review, Contini et al. 2017). Recently, large-scale brain activity has been associated to different personality characteristics (for review, Wagner et al. 2019). Our current work adds to this literature by showing that meaningful subjective differences can be connected to the classification of human brain responses already during the first 500 ms following stimulus presentation.

## Supplementary Material

nsae082_Supp

## Data Availability

The datasets generated and analyzed during the current study are available from the corresponding author on reasonable request.
