# Supplementary material for: Empathy enhances decoding accuracy of human neurophysiological responses to emotional facial expressions of humans and dogs
Source: Soc Cogn Affect Neurosci. 2024 Nov 7;19(1):nsae082. doi: 10.1093/scan/nsae082 (PMC11587893; doi:10.1093/scan/nsae082)
Supplement: nsae082_Supp [file nsae082_supp.zip › nsae082_Supp/scan-24-177-File008.pdf]

Supplementary material for:

Empathy enhances decoding accuracy of human neurophysiological responses to emotional facial expressions of humans and dogs

Miiamaaria V. Kujala[1,2,3,*], Lauri Parkkonen[3], Jan Kujala[1]

[1]Department of Psychology, Faculty of Education and Psychology, University of Jyväskylä, PO Box 35, FI-40014 University of Jyväskylä, Finland, [2]Faculty of Veterinary Medicine, PL 57, FI-00014 University of Helsinki, Finland, [3]Department of Neuroscience and Biomedical Engineering, Aalto University School of Science, P.O. Box 12200, FI-00076 Aalto, Finland

Supplementary Methods

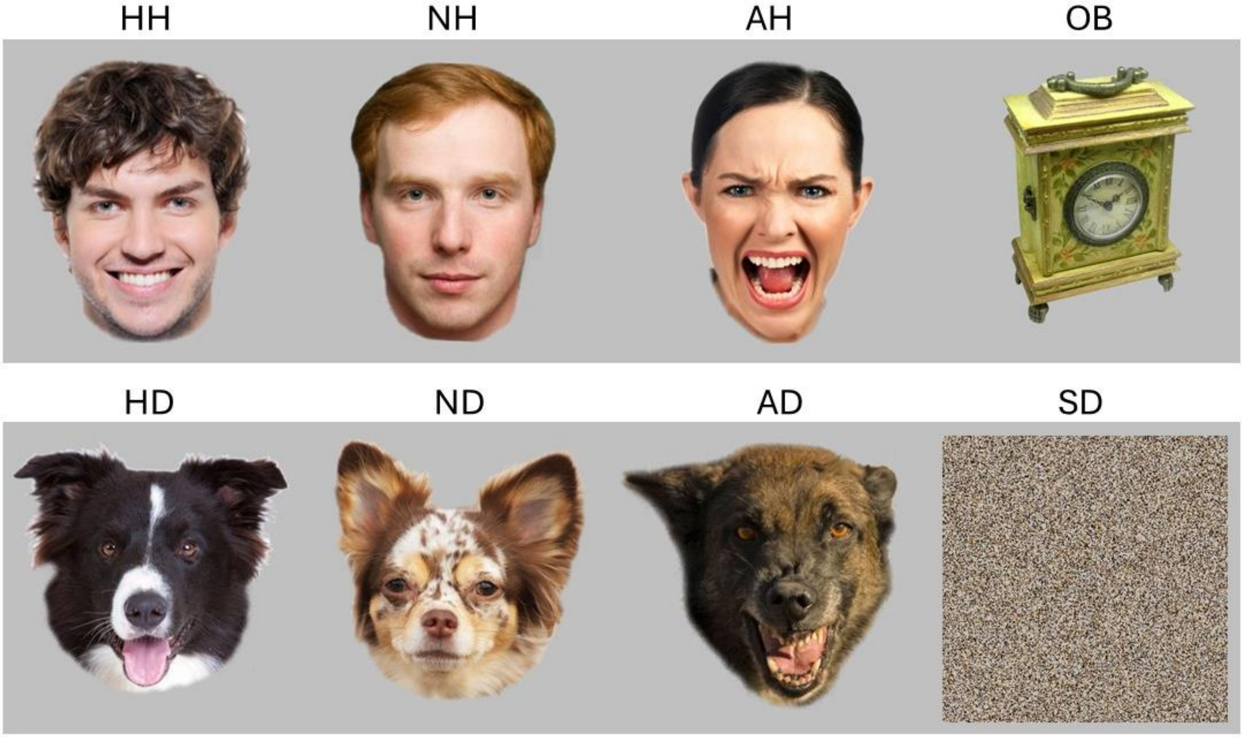

Figure S1. Examples of the stimulus categories. Top row: HH = happy human, NH = neutral human, AH = aggressive human, OB = object; Bottom row: HD = happy dog, NH = neutral dog, AD = aggressive dog, SD = phase-scrambled dog. Face stimuli have been described in Somppi et al Plos ONE 2016: https://doi.org/10.1371/journal.pone.0143047; objects in Stacy et al J Digit Imaging 1997: https://doi.org/10.1007%2FBF03168658; and phase-scrambled images in Kujala et al Plos ONE 2017: https://doi.org/10.1371/journal.pone.0170730).

Statistical analysis of the behavioral data

The variable distributions were explored with Shapiro-Wilk tests of normality and q-q plots; stimulus ratings were normally distributed, but not the response times nor classification accuracies. From the analyses including the behavioral data, responses of one subject were excluded due to a delay in

responding (> two standard deviations) and self-reported drowsiness during the behavioral measurement day.

To inform on the subjective evaluation of the stimulus categories as previously (Kujala *et al.*, 2017), the stimulus valence and arousal ratings were examined with repeated-measures ANOVA (species × expression); results were clarified with a priori planned comparisons with bootstrapping procedure (1000 samples, bias corrected and accelerated).

Thereafter, the subject response times to the valence and arousal ratings were examined with related-samples Friedman's two-way analysis of variance by ranks and the effect size was estimated by Kendall's coefficient of concordance; results were clarified with Dunn's pairwise comparisons.

All analyses were two-way with 95% confidence interval.

Source modeling of evoked responses

As an additional analysis, statistical comparison was performed for the mean absolute response strengths compared to baseline (-200 to 0 ms) activity for human faces (red) vs. dog faces (blue) in 100 ms long time windows, overlapping 50 ms, from 50 ms to 500 ms. All eight time windows were tested with a paired t-test at $p < 0.05$ (uncorr) and a cluster size threshold of ≥ 10 source points (Figure S3). These results show differences in neural responses to human and dog faces particularly in the parietal and temporal regions, suggesting that these regions potentially contribute to the perception of faces of different species. However, due to the lenient statistical threshold (uncorrected statistics), this interpretation would need to be verified in future studies with larger sample sizes.

Supplementary Results

Table S1. Subjective rating of each stimulus category, on a scale from 0 (lowest) to 7 (highest; mean ± SEM).

Rating	HH	HD	NH	ND	AH	AD	OB	SD
Valence	5.8 ± 0.1	5.1 ± 0.1	3.6 ± 0.1	3.8 ± 0.1	2.3 ± 0.2	1.9 ± 0.2	4.1 ± 0.1	3.9 ± 0.0
Arousal	4.1 ± 0.3	3.1 ± 0.2	2.1 ± 0.2	2.2 ± 0.2	5.2 ± 0.3	5.7 ± 0.2	1.2 ± 0.1	1.1 ± 0.1
Happiness	5.4 ± 0.3	3.9 ± 0.2	1.5 ± 0.1	1.6 ± 0.1	1.2 ± 0.1	1.3 ± 0.2	1.4 ± 0.1	1.0 ± 0.0
Sadness	1.2 ± 0.1	1.3 ± 0.1	2.2 ± 0.1	2.5 ± 0.3	1.6 ± 0.1	1.4 ± 0.1	1.1 ± 0.0	1.1 ± 0.1
Surprise	1.9 ± 0.2	1.9 ± 0.2	1.5 ± 0.1	1.5 ± 0.1	2.6 ± 0.3	2.4 ± 0.3	1.2 ± 0.1	1.0 ± 0.0
Disgust	1.0 ± 0.0	1.0 ± 0.0	1.7 ± 0.1	1.1 ± 0.0	3.5 ± 0.3	3.0 ± 0.2	1.0 ± 0.0	1.1 ± 0.1
Fear	1.1 ± 0.1	1.1 ± 0.0	1.6 ± 0.1	1.8 ± 0.2	2.4 ± 0.2	2.9 ± 0.3	1.0 ± 0.0	1.1 ± 0.1
Anger	1.1 ± 0.0	1.0 ± 0.0	1.5 ± 0.1	1.3 ± 0.1	4.7 ± 0.3	5.5 ± 0.3	1.1 ± 0.0	1.2 ± 0.2

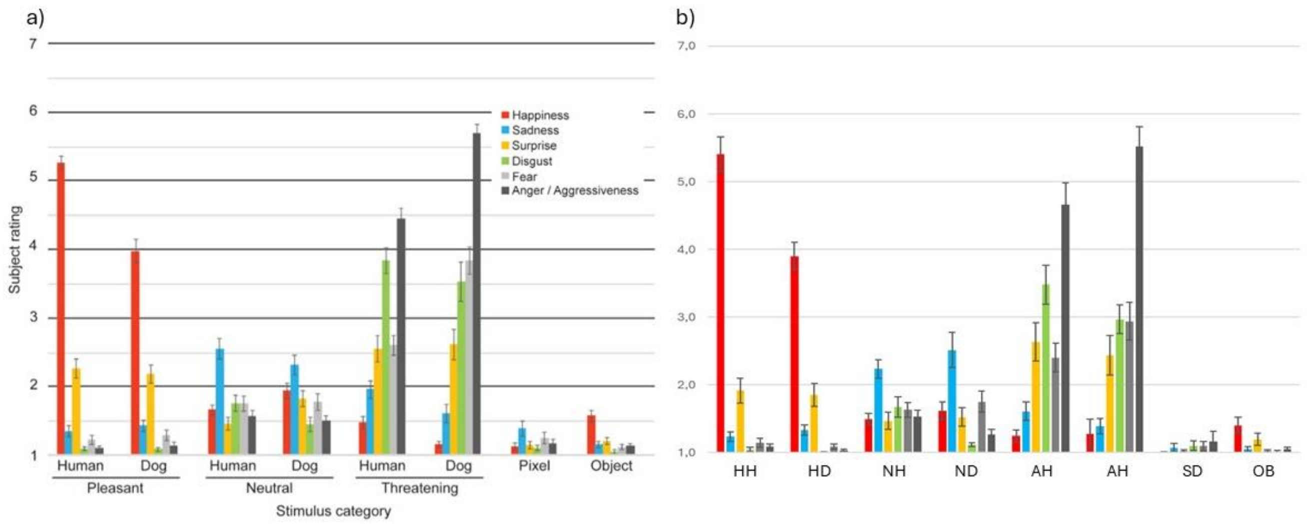

Figure S2. Behavioral rating of the discrete emotions in the stimulus images a) by the subjects of the previous study (Kujala et al Plos ONE 2017, figure replicated by the CC by 4.0 licence from https://doi.org/10.1371/journal.pone.0170730.g002; (mean ± SEM, n = 36); b) by the subjects of the current study (mean ± SEM, n = 15) depicted with the corresponding legend, color, and the order of categories. The current results (b) generally follow similar emotion ratings within a separate, smaller sample, especially considering happiness and anger/aggressiveness.

Rating valence. In valence ratings, main effects were found for species (human vs. dog; F(1) = 10.41, p = 0.007, ηp2 = 0.445) and expression (happy vs. neutral vs. aggressive; F(2) = 151.75, p < 0.001, ηp2 = 0.921), as well as interaction effect (species × expression; F(2) = 6.98, p = 0.004, ηp2 = 0.349).

Between species (human vs. dog expressions), HH was rated as more positive than HD (t(13) = 7.51, p < 0.001, d = 2.01, CI = 0.51–0.92), but neither NH vs. ND nor AH vs. AD differed in their valence ratings (NH vs. ND (t(13) = –1.55, p = 0.14, d = –0.41, CI = –0.28–0.04; AH vs. AD (t(13) = 1.37, p = 0.19, d = 0.36, CI = –0.19–0.89).

All human expressions differed in their rated valence; HH was rated as more positive than NH or AH, and NH as more positive than AH (HH vs. NH t(13) = 18.82, p < 0.001, d = 5.03, CI = 1.94–2.44; HH vs. AH t(13) = 12.99, p < 0.001, d = 3.47, CI = 2.94–4.11; NH vs. AH t(13) = 5.37, p < 0.001, d = 1.43, CI = 0.79–1.87).

Similarly, all dog expressions differed in their rated valence: HD was rated as more positive than ND or AD, and ND as more positive than AD (HD vs. ND t(13) = 12.95, p < 0.001, d = 3.46, CI = 1.12–1.57; HD vs. AD t(13) = 10.70, p < 0.001, d = 2.86, CI = 2.51–3.79; ND vs. AD t(13) = 8.01, p = 0.003, d = 2.14, CI = 1.32–2.29).

Rating arousal. For subject ratings of stimulus arousal, main effect was found for expression (F(2) = 90.67; $p < 0.001$; ηp2 = 0.875) but not species (F(1) = 2.26; p = 0.160; ηp2 = 0.146); an interaction effect (species × expression) was also found (F(2) = 20.65; $p < 0.001$; ηp2 = 0.614).

All human expressions differed in their rated arousal. AH was rated with highest arousal, followed by HH and NH (HH vs. NH t(13) = 6.34, $p < 0.001$, d = 1.69, CI = 1.30–2.64; HH vs. AH t(13) = –4.27, $p < 0.001$, d = –1.14, CI = –1.71– –0.56; NH vs. AH t(13) = –10.21, $p < 0.001$, d = –2.72, CI = –3.76– –2.44).

Also, rating of dog expressions followed the same pattern: AD was rated with highest arousal, followed by HD, and ND received the lowest arousal ratings (HD vs. ND t(13) = 3.75, $p < 0.001$, d = 1.01, CI = 0.39–1.46; HD vs. AD t(13) = –11.34, $p < 0.001$, d = –3.03, CI = –3.08– –2.09; ND vs. AD t(13) = –12.94, $p < 0.001$, d = –3.46, CI = –4.10– –2.93).

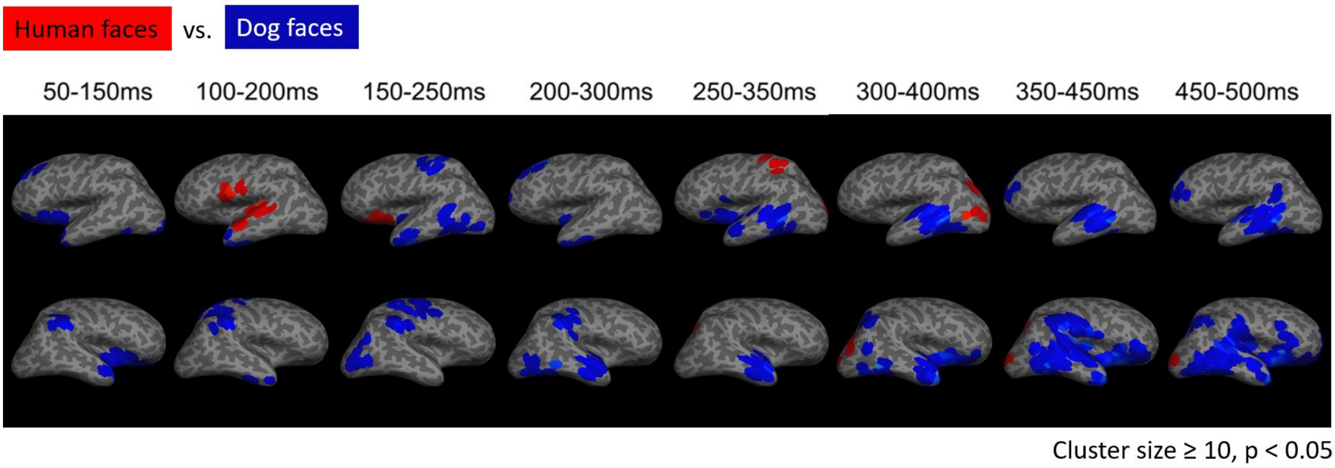

Figure S3. Statistical comparison of the response strengths for human faces (red) vs. dog faces (blue) in 100 ms long time windows, overlapping 50 ms, from 50 ms to 500 ms.

Table S2. Correlation between classification accuracy and trait-level empathy scores of EC (empathic concern) and PT (perspective taking). For the classification accuracy between the stimulus pairs of dog facial expressions, correlation to animal empathy scores animal EC (ani-EC) and animal PT (ani-PT) are also included.

**Method**	**Pair**	**EC (ani-EC)**			**PT (ani-PT)**		
		r_s	***p***	**CI**	***r_s***	***p***	**CI**
MEG	AD *vs.* HD	.329 (.093)	0.25 (0.75)	-.30–.84 (-.54–.67)	.377 **(.557)**	0.18 **(.038*)**	-.27–.73 (.01–.88)
EEG		.178 (-.040)	0.54 (0.89)	-.46–.83 (-.57–.52)	-.093 (.075)	0.75 (0.80)	-.68–.59 (-.53–.67)
MEG	AD *vs.* ND	.138 (-.195)	0.64 (0.51)	-.53–.77 (-.72–.58)	.156 (.178)	0.60 (0.54)	-.44–.66 (-.51–.85)
EEG		-.326 (-.350)	-0.33 (0.22)	-.83–.35 (-.85–.44)	-.056 (-.193)	0.85 (0.51)	-.62–.57 (-.78–.51)
MEG	AH *vs.* HH	.377	0.18	-.23–.84	.210	0.47	-.50–.74
EEG		.311	0.279	-.36–.80	**.486**	**0.078†**	-.29–.91

MEG	AH *vs.* NH	.266	0.359	-.33–.66	.323	0.260	-.22–.78
EEG		.213	.464	-.48–.77	.210	.471	-.51–.75
MEG	HH *vs.* NH	.264	.361	-.26–.67	.278	.336	-.25–.71
EEG		**.732**	**.003****	.33–.91	**.672**	**.008****	.19–.94

* $p < 0.05$; ** $p < 0.01$; *** $p < 0.001$; † $p < 0.1$.

Table S3. Correlation between classification accuracy and respective response times for the valence and arousal of each of the classification pairs.

	Classification (Pair 1 vs. Pair 2)	Response time for Pair 1			Response time for Pair 2		
		r_s	p	CI	r_s	p	CI
**Valence**	AH *vs.* HH	**0.61**	**0.021***	0.10–0.87	0.34	0.24	-0.14–0.71
	HH *vs.* NH	-0.25	0.38	-0.67–0.28	0.41	0.15	-018–0.76
	AH *vs.* NH	**0.52**	**0.052†**	0.06–0.84	0.12	0.69	-0.59–0.72
**Arousal**	AH *vs.* HH	0.39	0.18	-0.19–0.75	**0.57**	**0.032***	0.04–0.85
	HH *vs.* NH	0.12	0.68	-0.49–0.69	-0.35	0.22	-0.75–0.16
	AH *vs.* NH	0.17	0.55	-0.40–0.65	-0.34	0.23	-0.83–0.19

* $p < 0.05$; ** $p < 0.01$; *** $p < 0.001$; † $p < 0.1$.

EEG machine learning analysis

For the EEG data, discrimination of event-related brain responses over the whole 0–500 ms time window was significant (p < 0.05, corrected for multiple comparisons) primarily between faces of different species or faces vs. objects/scrambled images (see Figure S4 bottom). The discrimination between different dog and human expressions, in turn, was significant only in individual subjects. The classification accuracy of all face categories *vs.* scrambled was 92% (across-subjects range 65–100%) and for object vs. scrambled 87% (range 65–96%). Discrimination of human faces from dog faces with comparable valence yielded accuracy of 77% (range 55–90%). Classification accuracy across subjects in different comparisons of species, emotions or objects for EEG data is depicted in the top part of Figure S4. The classification accuracy between the different facial expressions was markedly similar for both species, with small differences across the different comparisons. For both species, the best discrimination was seen between angry and neutral faces. Between dog expressions, the classification accuracies were as follows: AD vs. ND 59% (across-subject range 50–76%), AD vs. HD 56% (range 49–60%), and HD vs. ND 54% (range 46–66%). Between human expressions, the accuracies were the following: AH vs. NH 58 % (range 49–72%), AH vs. HH 54% (range 44–68%), and HH vs. NH 53% (range 46–61%).

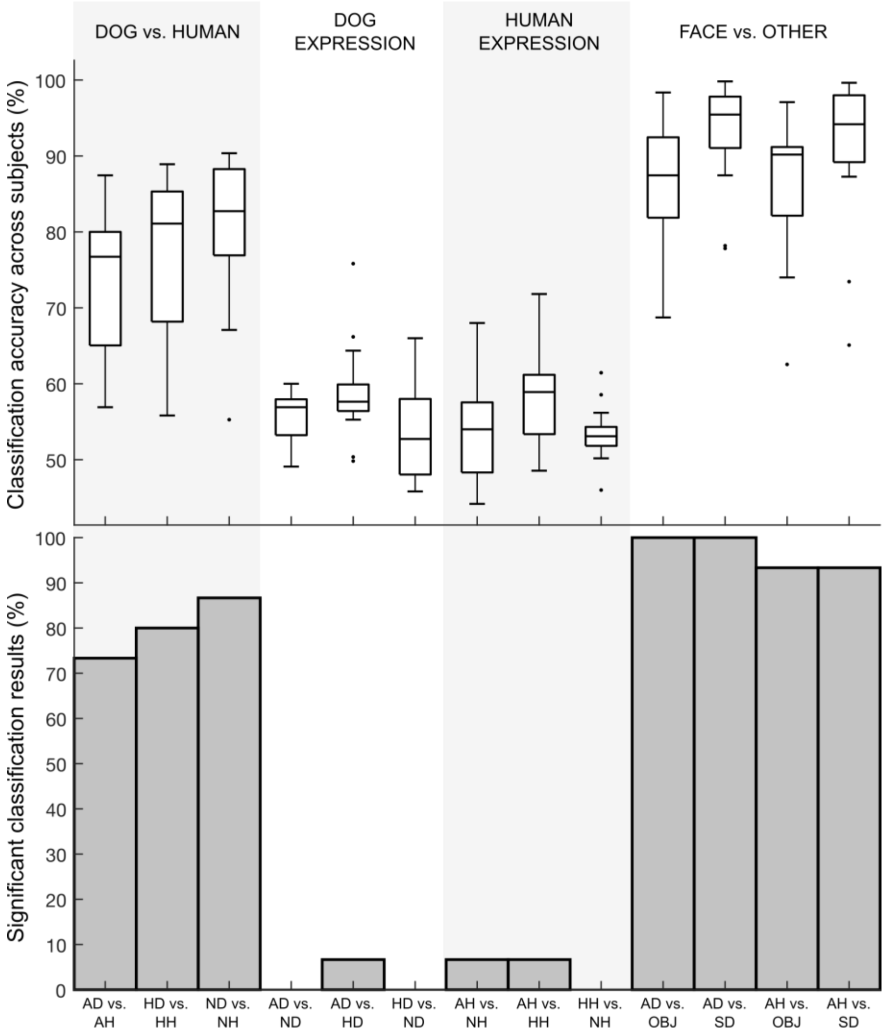

Figure S4. Results from the EEG classification over the whole 0–500 ms time window. Top: Classification accuracy across subjects as a boxplot in pairwise comparisons of dog vs. human; dog facial expression; human facial expression; and faces (aggressive dog/human) vs. objects or scrambled images. Bottom: Percentage of subjects with significant classification results in the pairwise comparisons. The pairs are given below the figure.
